# Fluoromicrometry reveals minimal influence of tendon elasticity during snake locomotion

**DOI:** 10.1242/jeb.249259

**Published:** 2025-03-03

**Authors:** Jessica L. Tingle, Kelsey L. Garner, Henry C. Astley

**Affiliations:** Department of Biology, University of Akron, Akron, OH 44325, USA

**Keywords:** Biomechanics, Functional morphology, Lateral flexion, Range of motion, Squamates

## Abstract

Multiarticular muscle systems are widespread across vertebrates, including in their necks, digits, tails and trunks. In secondarily limbless tetrapods, the multiarticular trunk muscles power nearly all behaviors. Using snakes as a study system, we previously used anatomical measurements and mathematical modeling to derive an equation relating multiarticular trunk muscle shortening to postural change. However, some snake trunk muscles have long, thin tendinous connections, raising the possibility of elastic energy storage, which could lead to a decoupling of muscle length change from joint angle change. The next step, therefore, is to determine whether *in vivo* muscle shortening produces the postural changes predicted by mathematical modeling. A departure from predictions would implicate elastic energy storage. To test the relationship between muscle strain and posture *in vivo*, we implanted radio-opaque metal beads in three muscles of interest in four corn snakes (*Pantherophis guttatus*), then recorded X-ray videos to directly measure muscle shortening and vertebral column curvature during locomotion. Our *in vivo* results produced evidence that elastic energy storage does not play a substantial role in corn snake lateral undulation or tunnel concertina locomotion. The ability to predict muscle shortening directly from observed posture will facilitate future work. Moreover, the generality of our equation, which uses anatomical values that can be measured in many types of animals, means that our framework for understanding multiarticular muscle function can be applied in numerous study systems to provide a stronger mechanistic understanding of organismal function.

## Teaser

**Summary:** Investigation of the relationship between muscle strain and posture in corn snakes *in vivo* using fluoromicrometry reveals a strong, predominately linear relationship between muscle shortening and vertebral column curvature, as predicted by mathematical modeling.

## INTRODUCTION

The geometric relationship between muscles and joints has a powerful impact on functional outcomes. Although the influence of lever arms on monoarticular muscles has been understood at least since the time of Aristotle (Aristoteles and Nussbaum, 1985), and our understanding of more complex biarticular muscles has improved in recent decades (van Ingen Schenau, 1989; van Ingen Schenau et al., 1994), the mechanics of multiarticular muscles (crossing more than two joints) remain sparsely investigated (Astley, 2020; Tingle et al., 2023). Multiarticular muscle systems serve important functions across vertebrates, including propulsion and/or stabilization during locomotion (Ritter, 1992; Gramsbergen et al., 1999; O'Reilly et al., 2000; Cromwell et al., 2001; Saunders et al., 2004; Schilling and Carrier, 2010; Omura et al., 2015). They are particularly important in the many tetrapods that have secondarily evolved body plans with reduced or absent limbs, as these species rely almost completely on multiarticular trunk muscles to power movement (Jayne, 1988a,b; Wiens et al., 2006; Bergmann et al., 2020; Tingle et al., 2024).

We previously used anatomical measurements and an explicitly multiarticular mathematical modeling approach to predict the effects of muscle architecture on torque production and postural change in corn snakes (Tingle et al., 2023). As the next step towards a more complete understanding of multiarticular muscle mechanics, we need to determine whether *in vivo* muscle contraction produces the postural changes predicted by mathematical modeling. This modeling provides a basis for the hypothesis that muscle strain (relative length change) is directly predictable from vertebral curvature. However, some trunk muscles have long, thin tendinous connections, raising the possibility of an alternative hypothesis: that elastic energy storage could lead to a decoupling of muscle length change from joint angle change. Elastic energy storage in spring-like tendons is common in limbed tetrapods – with the tendons acting as springs, they can achieve greater power than they would through muscle activity alone (i.e. elastic power amplification or elastic catapult mechanisms) (Peplowski and Marsh, 1997; Roberts et al., 1997; Roberts and Marsh, 2003; Roberts and Azizi, 2011; Astley and Roberts, 2012).

**Fig. 1.**
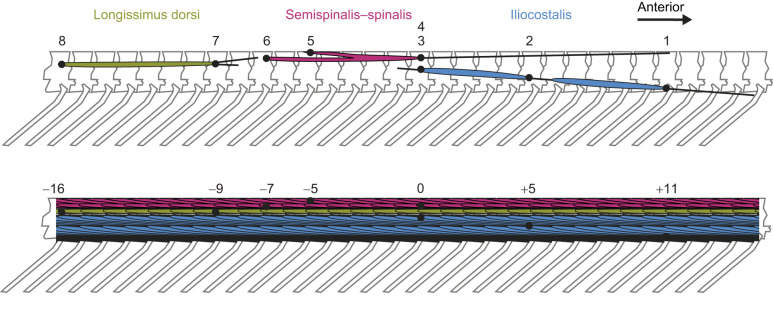
**Locations of bead implantation.** Beads track distances between key locations in the longissimus dorsi, semispinalis, spinalis and iliocostalis muscles. The top panel shows a single muscle of each type for clarity, whereas the bottom panel shows the tracts of overlapping muscles. For the purposes of our analyses, we numbered beads anterior to posterior (as indicated in the top panel), with beads 1–3 embedded in the iliocostalis, beads 4–6 in the semispinalis–spinalis (4–5 indicates the span of the spinalis, which is the dorsal fork of the muscle belly, and 4–6 indicates the semispinalis, which is the ventral fork), and beads 7 and 8 in the longissimus dorsi. Location 0 in the bottom panel represents the site of the first surgical incision, where we implanted beads 3 and 4; distances between other beads are noted in numbers of vertebrae anterior (positive numbers) or posterior (negative numbers) to the first incision site.

Although elastic energy storage could occur, we did not expect to find evidence for this alternative hypothesis in most muscles of interest. In muscle–tendon units that show significant elastic effects, the tendon is often considerably longer than the muscle (Biewener and Roberts, 2000). In corn snakes, only one epaxial muscle has an especially long, thin tendon that could be capable of appreciable elastic energy storage: the semispinalis–spinalis anterior tendon spans 11 vertebrae in corn snakes (Tingle et al., 2023; Fig. 1) and >30 vertebrae in some species (see Appendix S4 from Tingle et al., 2017). However, its position very near to the midline does not give it good leverage for lateral flexion (Tingle et al., 2023), so it is unlikely to undergo much strain during the lateral bending that characterizes most forms of snake locomotion. Without undergoing such strain, it would not have the opportunity to store much elastic energy. The iliocostalis has better leverage for lateral flexion, but its longest tendon (the anterior tendon) is much shorter, spanning only four vertebrae in corn snakes (Tingle et al., 2023; Fig. 1).

To test our competing hypotheses, we used fluoromicrometry (Camp et al., 2016) to measure muscle contraction and cineradiography to measure vertebral column curvature in living snakes, then compared *in vivo* results with the expectation based on our previously derived equation (Tingle et al., 2023). If fluoromicrometry demonstrates a linear relationship between vertebral column curvature and muscle strain, then muscle strain is directly predictable from vertebral bending. If joint motion and muscle strain are decoupled, with joint motion occurring without corresponding muscle length change and vice versa (Roberts et al., 1997), then elastic energy storage will be implicated.

## MATERIALS AND METHODS

### Animal acquisition and care, implantation of radio-opaque beads

Four wild-caught adult corn snakes, *Pantherophis guttatus* (Linnaeus 1766), were purchased from a commercial provider (Glades Reptiles, Bushnell, FL, USA) and kept under University of Akron IACUC protocol no. 16-08-16-ASD. These individuals ranged in mass from 520 to 540 g and in snout–vent length from 104 to 120 cm. They were kept individually in 61×122×38 cm PVC cages with aspen substrate prior to bead-implantation surgery (detailed below) and paper towel substrate after surgery. A radiant heat panel provided a temperature gradient from 29 to 23°C. They were fed pre-killed adult mice biweekly.

To mark key locations in epaxial muscles, we performed surgeries to implant radio-opaque tantalum beads measuring 0.8 mm in diameter (X-medics, Frederiksberg, Denmark). Surgeries followed aseptic technique and were performed between 9 and 18 May 2023, with snakes fasted for 5–14 days prior to surgery. Anesthesia was induced in a chamber filled with 5% isoflurane, and snakes were maintained on 5% isoflurane via intubation during surgery. They were also given carprofen (4 mg kg^−1^) during surgery for pain relief. Immediately after surgery, snakes were closely monitored in an incubation chamber at 28°C until they recovered from anesthesia, as indicated by a return to basic locomotor function. Post-surgery care was provided by University of Akron Vivarium staff, which included the administration of 2 mg kg^−1^ of carprofen every 48 h or as needed. Incision sites were monitored for proper healing and removal of sutures leading up to locomotor trials.

For each snake, we made five small incisions in the right side of the body at approximately the midpoint between the snout and the cloaca, used a syringe with a plunger to inject the beads, and then used Vetbond (3M, St Paul, MN, USA) to seal the small hole made by the syringe and 4-0 nylon monofilament sutures to close the incision. Traditionally, fluoromicrometry and sonomicrometry involve the implantation of beads or crystals inside a single muscle but between and parallel to muscle fascicles (Camp et al., 2016; Roberts and Dick, 2023). However, the anatomy of snake muscles requires a modification to bead implantation practices. Individual muscles occur in overlapping tracts (Fig. 1, bottom) in which the individual muscles are so closely apposed that clearly separating successive muscle tracts would cause unacceptable mechanical damage, requiring us to use anatomical knowledge from prior dissections for placement. All segments of a given muscle bundle (longissimus dorsi, iliocostalis, semispinalis–spinalis) are tightly wrapped together in a layer of connective tissue, keeping the segments (and the beads embedded in them) separate from those of other muscle types. Because the muscles are long with cross-sectional areas less than that of the beads, and their multiarticular nature leads to many overlapping segments of a given type of muscle, the beads were placed between two overlapping segments of the muscle of interest rather than inside a single muscle segment, a situation analogous to bead placement between fascicles of larger muscles.

Pairs of beads marked three epaxial muscles: the longissimus dorsi, the iliocostalis and the semispinalis–spinalis (Fig. 1). Each bead pair was placed such that the number of vertebrae in between them equaled approximately the number of vertebrae spanned by the muscles into which they were inserted (7 vertebrae between the two beads marking the longissimus dorsi, 11 for the iliocostalis, 7 for the semispinalis portion of the semispinalis–spinalis, and 5 for the spinalis portion). For the iliocostalis, we implanted an additional bead near the middle of the muscle–tendon unit's span to mark the location of the medial tendon (bead 2; Fig. 1). We also implanted three beads into the semispinalis–spinalis: one to indicate the anterior end of the muscle belly (bead 4), one to mark the posterior end of the spinalis (the dorsal fork of the muscle belly; bead 5) and one to mark the posterior end of the semispinalis (the ventral fork of the muscle belly; bead 6) (Fig. 1).

We confirmed bead placement via post-surgical imaging with cinefluoroscopy, which clearly showed that the beads had remained in position after 4–5 weeks of healing. In nearly all cases, beads were separated by the intended number of vertebrae (deviating no more than 2 vertebrae from the intended number), and they were located at the approximately intended distances from the vertebral column in both the medio-lateral and dorso-ventral planes. An exception was the bead pair marking the spinalis in one individual (snake 1), which were separated by only 2.5 vertebrae instead of the intended 5 vertebrae. Deviation from the intended bead placement did not seem to have a major impact on the results, so figures include data for that muscle (clearly marked in its own panel).

### Biplanar videofluoroscopy of locomotor trials

**Fig. 2.**
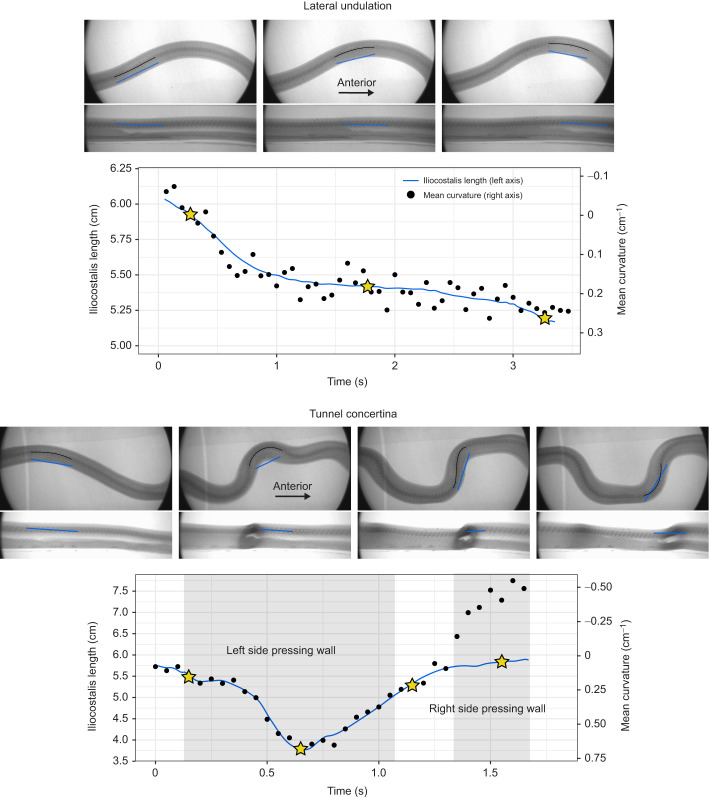
**Iliocostalis muscle length versus body posture in representative trials for lateral undulation and tunnel concertina locomotion.** The plots display iliocostalis muscle length alongside mean curvature of the section of vertebral column spanned by the iliocostalis over the course of the trial. Muscle length is displayed as blue lines with *y*-axis values on the left, and was calculated as the distance between the first and third radio-opaque beads from videos digitized in XMALab. Curvature is displayed as black points with *y*-axis values on the right (with negative values on top, due to our sign convention), and was calculated from still images in ImageJ. Curvature of 0 cm^−1^ indicates a straight body posture (no curvature); negative curvature values indicate a curve to the left (radio-opaque beads on the outside of the curve), whereas positive curvature values indicate a curve to the right (radio-opaque beads on the inside of the curve). Stars indicate frames for which still images are displayed above the plots (ventral views on top, lateral views underneath those). Colors in the images correspond to those in the plots: blue lines on the images indicate the distance between the beads (our proxy for muscle length), and black lines on the images indicate the curve of the vertebral column spanned by the two beads. Shaded areas of the tunnel concertina plot indicate approximate times when the body was pressing against the tunnel wall. Tunnel walls are barely visible, as the tunnel was made from foam that is nearly transparent to X-rays.

For locomotor trials, we used one trackway to elicit tunnel concertina locomotion and two to elicit lateral undulation (Fig. 2). All trackways were 135 cm long and built out of extruded polystyrene (XPS) rigid foam insulation boards (Owens Corning Foamular 150). The concertina tunnel was 8.5 cm wide, a width chosen based on the 7.5 and 10 cm wide tunnels previously used to elicit concertina locomotion in a similarly sized, related species, *Pantherophis obsoletus* (Jayne, 1988a). Both of the lateral undulation setups were 20 cm wide with walls on the sides, providing enough space for adult corn snakes to slither unhindered. The walls had blocks (made of the same XPS foam as the trackway) protruding from them, interspersed with block-free spaces to create alternating push points. In one lateral undulation setup, 10 cm long blocks alternated with 10 cm long spaces. In the other setup, 10 cm long blocks alternated with 20 cm long spaces. In both setups, the right and left sides of the trackways had blocks out of phase by 180 deg, i.e. staggered so that a block on one wall would face a blank space on the other wall. The blocks were 5 cm wide, such that 10 cm in the middle of the trackway was totally clear. The two lateral undulation setups were intended to encourage different amounts of curvature during locomotion.

We conducted locomotor trials slightly more than 5 weeks post-surgery using the biplanar cineradiography system at Northeast Ohio Medical University (GE 9400 C-Arms). We tested all snakes first on the lateral undulation track with small spacing, followed by the concertina tunnel, and finally on the lateral undulation track with large spacing. For each track type, we tapped the snake's tail to encourage it to move down the trackway for 3–4 trials. Snakes were kept in an incubator at 28°C between trials on different tracks. The ambient room temperature and the trackway surfaces were kept at 24–25°C. Trials were recorded with high-speed videofluoroscopy (XC1M, XCitex, Cambridge, MA, USA), using two cameras synchronized with a 40 MHz signal from a DAQ card in the computer. For lateral undulation trials, we set both cameras to 150 frames s^−1^, 70 kV and 4.7 mA. These settings prevented motion blur while still providing high-contrast imaging of beads and bone for snakes moving at typical lateral undulation speeds. For concertina locomotion, which is typically slower than lateral undulation, we set both cameras to 100 frames s^−1^ and 4.7 mA, with the laterally oriented camera at 70 kV and the ventrally oriented camera at 60 kV. Image intensifiers were positioned perpendicular and adjacent to each other, and trackways were positioned a few centimeters above the ventral image intensifier and almost abutting the lateral one. Videos and associated files are available on XMAPortal (https://xmaportal.org/webportal/, under study name ‘Snake Fluoromicrometry’ and study identifier XMA5).

### Data processing

To calculate 3D coordinates of beads over the course of each trial, we digitized videos in XMALab (Brainerd et al., 2010; Knörlein et al., 2016), using undistortion grids and a custom calibration cube to undistort and calibrate the field of view. The markers' mean reprojection errors ranged from 0.36 to 1.89 pixels (mean±s.d.: 0.82±0.31 pixels) across the trials used in our analysis. We smoothed the *x*, *y* and *z* values independently from each other using a cubic spline with *s*=0.01, implemented with the splrep() function in the SciPy package (Virtanen et al., 2020) in Python version 3.9.7. From the smoothed 3D coordinates, we calculated straight-line, 3D distance [√(*x*^2^+*y*^2^+*z*^2^)] between bead pairs of interest, representing the longissimus dorsi (beads 7 and 8), the iliocostalis (beads 1 and 3), the semispinalis portion of the semispinalis–spinalis (beads 4 and 6), and the spinalis portion of the semispinalis–spinalis (beads 4 and 5) (Fig. 1). The blue lines on still images in Fig. 2 represent these straight-line distances between bead pairs. The straight-line distances do not exactly correspond to the shape of the muscle at times when the body is very curved, as made clear in the concertina image where the straight line is not fully inside the body. However, they are a reasonable approximation of the muscle's length, with >90% accuracy for the most extreme body curvatures observed in this study, and ∼99% accuracy for curvatures more typical of lateral undulation.

**Fig. 3.**
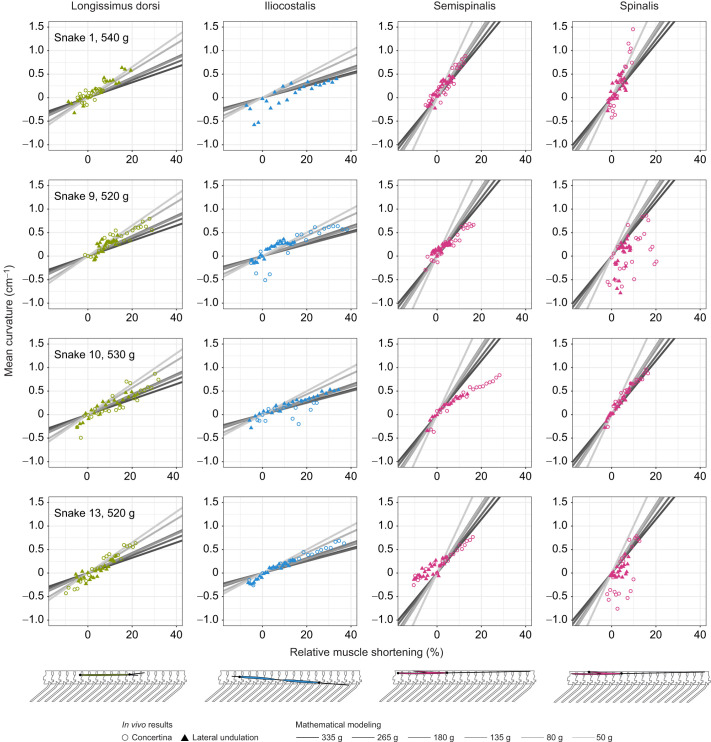
**Relationship between mean vertebral column curvature and muscle strain during lateral flexion.** Plots show vertebral column curvature in the lateral plane when a given muscle shortens by some percentage of its resting length. Points correspond to *in vivo* results from the present study for snakes (ID numbers: 1, 9, 10 and 13) performing both lateral undulation (triangles) and tunnel concertina locomotion (circles). For each individual, we include data from all trials for a given locomotor mode (up to three trials). For snake 1's iliocostalis muscle, we have data only for lateral undulation because the snake's body was pressed against the tunnel wall during that snake's only concertina trial, deforming the iliocostalis. Gray lines correspond to the mathematical modeling results for six preserved snakes of different sizes from a previous study (Tingle et al., 2023). They are included to highlight that slopes calculated for *in vivo* data in the present study do not strongly deviate from those predicted by mathematical modeling. Steeper slopes indicate more postural change when the muscle contracts, as measured by the mean curvature of the section of vertebral column spanned by the muscle of interest. Shallow slopes indicate that the muscle effects less postural change but greater torque.

We then calculated curvature of the snake's vertebral column in a subset of frames, selected to represent the range of curvature from most concave to straight to slightly convex, with even sampling across this range of curvature values (Fig. 3). Because we were interested in curvature in the horizontal plane, we used only the camera providing a ventral view. Although snakes may lift their bodies during both lateral undulation and tunnel concertina (Hu et al., 2009; Marvi and Hu, 2012), their motions are primarily in the horizontal plane, and we observed minimal lifting in the vertical plane for these trials. For each muscle of each snake, we chose a subset of 20–25 frames from the digitized videos for each locomotor mode (lateral undulation and tunnel concertina), pooling all trials within each locomotor mode. These frames included one where the muscle had reached its most contracted state for a given locomotor mode, one where the muscle was approximately resting length (i.e. the section of vertebral column spanned by the muscle was straight), with 18 evenly spaced frames in between, and up to 5 evenly spaced frames with the muscle stretched slightly beyond resting length. For these frames, we manually digitized the midline of the section of vertebral column spanned by the muscle of interest, using the line segment tool in the Fiji distribution of ImageJ 1.53t, Java 1.8.0_172 (Schindelin et al., 2012). We then applied a custom macro in ImageJ (see Supplementary Materials and Methods) to smooth the backbone curve and output *x* and *y* coordinates for 100 evenly spaced points along it. Finally, we calculated curvature for each segment of the smoothed backbone curve using Eqn 1, which approximates derivatives via finite differences in *x* and *y* along the snake's body length:
(1)

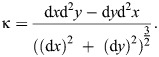
We then averaged the curvatures of the segments for a given time frame to get the overall curvature for the section of vertebral column in that frame. We subsequently converted curvature units from inverse pixels to inverse centimeters using a rigid object of known length that was in the camera view for some trackways.

To test whether muscle length is directly predictable from posture, the main goal of this paper, we directly examined the relationship between curvature (κ) and relative muscle shortening as a percentage of its length when the body posture is straight (analysis described below). However, to facilitate comparison with previously published results (Jayne, 1988a,b; Morinaga and Bergmann, 2019), we also estimated mean intervertebral joint angle from mean curvature, using Eqn 2:
(2)

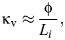
where *L_i_* equals the length of each vertebra and φ equals the angle between each pair of vertebrae in radians (Tingle et al., 2023). Because our videos did not allow us to directly measure the length of individual vertebrae, we used allometric equations from our previous paper (Tingle et al., 2023) to first estimate vertebral length of the snakes in the present study based on body size.

### Evaluation of our mathematical model with *in vivo* data

We used a series of statistical models to evaluate whether vertebral column curvature and muscle strain showed a linear relationship, as predicted by our mathematical model. We evaluated lateral undulation and concertina trials separately. During portions of some concertina trials, the muscle of interest was pressed against the wall of the tunnel, leading to deformation of the muscle via external force. Because these data points are inapplicable for the evaluation of our model, we removed them prior to analyses. For each muscle of each snake, we fitted a series of four models for mean body curvature, calculating Akaike's information criterion corrected for small sample size (AICc) for each to facilitate model comparison. These models included an intercept-only model, an ordinary least squares (OLS) regression on relative muscle shortening, a regression on muscle shortening plus muscle shortening squared, and finally a regression that included linear, squared and cubed terms. Prior to squaring and cubing relative muscle shortening, we standardized each value (subtracted the mean of all relative shortening values for that muscle of that snake, and then divided by the standard deviation) to reduce the correlation of the squared and cubed terms with the original measure of relative shortening (orthogonal polynomial). These tests included data points from up to three trials per snake, but there is no physical or physiological reason to expect the relationship between muscle shortening and body curvature to differ across trials. We used the SAS Multtest procedure to control for positive false discovery rate (pFDR) for the squared and cubed terms across all models. This procedure uses the distribution of *P*-values to estimate the ratio of true null tests to total tests, and the *q*-value provides a measure of significance in terms of the false discovery rate (Storey, 2002, 2003; Storey and Tibshirani, 2003; Storey et al., 2004). We did not include *P*-values from the linear term for relative muscle shortening in pFDR because of the certainty that it would have a strong relationship with body curvature. At *q*=0.05, all *P-*values under 0.05 remained statistically significant.

## RESULTS

As expected, we found that relative muscle shortening significantly predicted mean vertebral column curvature in all muscles for all snakes in both lateral undulation and tunnel concertina trials, and that statistical models with muscle shortening as a predictor outperformed the intercept-only models in all cases (Table S1). Examining the statistical models with only a linear predictor term (mean curvature∼relative muscle shortening), we found that for the 16 lateral undulation tests (4 muscles×4 snakes), mean±s.d. *r*^2^=0.73±0.19, indicating that our linear approximation of relative muscle shortening by itself explains 73% of the variance in curvature, on average. For the 15 tunnel concertina tests (one snake was missing data for the iliocostalis muscle), mean±s.d. *r*^2^=0.79±0.21, indicating that relative muscle shortening by itself explains 79% of the variance in curvature, on average.

In some cases, we found evidence for statistically significant squared and/or cubed terms in the statistical models after controlling for pFDR at *q*=0.05. In six of the 16 lateral undulation tests, the linear model was preferred over more complex models (ΔAICc<−2) or was indistinguishable based on AICc (−2≤ΔAICc≤0). In six of the lateral undulation tests, a model that also included a squared term was preferred, and in four, a model including squared and cubed terms was preferred. The linear model was preferred in 10 of 15 tunnel concertina tests, whereas a model with a squared term was preferred in two tests, and a model with squared and cubed terms was preferred in three tests (Table S1).

**Fig. 4.**
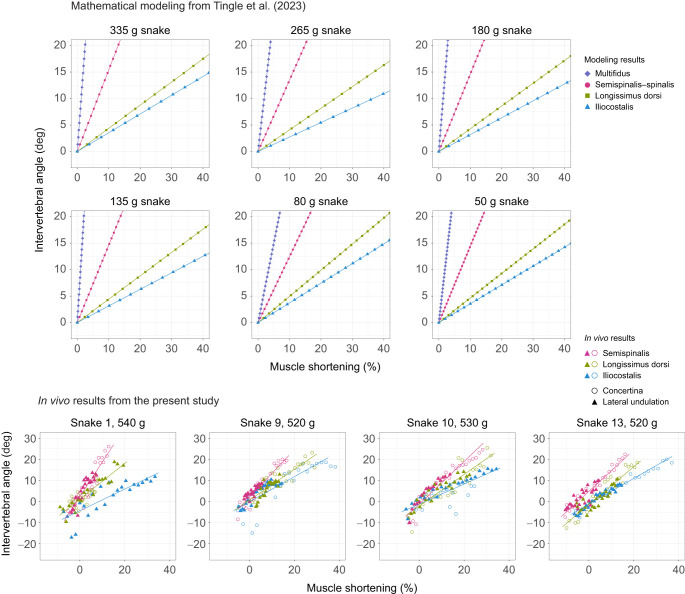
**Relationship between mean intervertebral angle and muscle strain during lateral flexion.** Plots of mean intervertebral angle (deg) instead of curvature (cm^−1^) versus muscle strain, with each plot showing all the muscles for a given-sized snake to facilitate comparison of the different muscles. The mathematical modeling plots correspond to fig. 4 from Tingle et al. (2023), except that the earlier figure presents results for dorsoventral flexion, whereas this figure presents results for lateral flexion. Mathematical modeling predicts higher slopes for the semispinalis–spinalis and the multifidus than for the longissimus dorsi and iliocostalis, indicating that the former two muscles contribute relatively more to postural change whereas the latter two contribute relatively more to torque. *In vivo* results match that prediction. Note that the *y*-axis ranges differ slightly between the six plots at the top, which display results from our previous mathematical models, and the four plots at the bottom, which display *in vivo* data from the present study. Note also that the mathematical modeling plots include a line for the multifidus muscle, for which we do not have *in vivo* data. To calculate mean intervertebral angle for *in vivo* data, we had to first estimate the length of individual vertebrae based on allometric equations from Tingle et al. (2023), as vertebral lengths could not be measured from videos.

As predicted from mathematical models, estimated slopes were highest for the semispinalis, indicating that of the muscles examined, it generates more postural change but less torque for a given amount of muscle strain during lateral flexion, whereas the slope was lowest for the iliocostalis, indicating that the iliocostalis generates the most torque at the expense of postural change for a given muscle strain (Fig. 4). The relationship was generally noisier for the spinalis than for the other muscles (Fig. 3; Table S1), likely due to the forked anatomy of the semispinalis–spinalis, in which the spinalis portion of the muscle belly runs off-axis to the long anterior tendon.

Snakes curved their bodies more tightly during tunnel concertina than during lateral undulation, with intervertebral angles reaching ∼20 deg during tunnel concertina and only ∼10 deg during lateral undulation (Fig. 4). The fastest shortening speeds during tunnel concertina were 2.6 resting muscle lengths (*L*) per second for the longissimus dorsi, 1.6 *L* s^−1^ for the iliocostalis and 2.5 *L* s^−1^ for the semispinalis. The fastest shortening speeds during lateral undulation were 1.5 *L* s^−1^ for the longissimus dorsi, 0.8 *L* s^−1^ for the iliocostalis and 1.5 *L* s^−1^ for the semispinalis.

## DISCUSSION

We found strong evidence that muscle shortening predicts body curvature, and no evidence that elastic energy storage plays a substantial role in corn snake lateral undulation or tunnel concertina locomotion, supporting our previous mathematical modeling (Tingle et al., 2023). If elastic energy storage did play a major role, then we would clearly see some amount of decoupling between muscle shortening and changes in body curvature, in which the muscle would sometimes shorten without the expected change in curvature because tendon stretch would offset the muscle shortening (Astley and Roberts, 2012; Konow et al., 2015). We did not see such a pattern for any of the muscles we examined (Fig. 3). It is particularly striking that we did not find evidence for tendon stretch even during tunnel concertina, which involves notoriously high forces: corn snakes have been measured pushing on tunnel walls with force equaling about twice their body weight in level tunnels, and up to nine times their body weight in inclined tunnels (Marvi and Hu, 2012).

While it remains possible that the tendons stretch substantially in other high-force behaviors such as cantilevered gap bridging (Jayne and Riley, 2007) or constriction (Moon, 2000; Moon and Mehta, 2007), our results show that we can infer muscle shortening from body posture in corn snakes during two common locomotor behaviors, and likely for other low-force behaviors as well. These results may also apply to other snake species, particularly those with tendon lengths equal to or shorter than those of corn snakes. Although the literature on comparative snake muscular anatomy is sparse, systematic research on the semispinalis–spinalis complex (Jayne, 1982; Tingle et al., 2017) and descriptions of other muscles across a diversity of snake families (Gasc, 1974, 1977, 1981) suggest that corn snake tendon lengths are fairly typical for caenophidian snakes. Thus, inferring muscle length from body curvature may be plausible across a wide range of snake species, an ability that will facilitate future research on muscle function during snake locomotion.

Although we did not find evidence for elastic energy storage during lateral undulation or tunnel concertina, we found that the relationship between muscle shortening and postural change was not always purely linear. In almost half of cases (4 muscles×4 snakes×2 locomotor modes), squared and/or cubed muscle shortening had a statistically significant association with curvature. It was not consistent which of the more complex models was favored (one with squared versus squared+cubed terms), and the estimated effects of these predictors had inconsistent signs, with some positive and others negative (Table S1). Given these inconsistencies, we cannot speculate why squared and/or cubed terms are significant for some trials, and we do not think that these more complex models provide useful physical information about snake muscle function.

We recorded several instances of outliers where the snake's posture for a given muscle length did not match the prediction, with more outliers for the iliocostalis than for other muscles (see Materials and Methods). These outliers all occurred during concertina trials at times when the section of body containing the muscle was at least partially pressed against the wall of the tunnel (Fig. 2). The outliers may therefore result from two potential causes, which are not mutually exclusive: first, the lateral position of the iliocostalis may result in physical compression of the muscle itself (see fig. 1B in Tingle et al., 2023), and second, the rib motion observable during concertina locomotion (Fig. 2) may alter the location of the anterior attachment of the muscle, consequently changing its lever arm. These possibilities invite caution for future studies that involve inference of muscle strain from external posture when the body wall deforms substantially, but also raise the interesting possibility that muscle function may be altered by active or passive motion of the ribs.

Our results and their implications broadly agree with the prior literature on snake locomotion. Observed ranges of muscle shortening (Fig. 3) correspond well to changes in sarcomere length in specimens fixed in straight and maximally bent postures (Jurestovsky et al., 2022), while the observed curvatures, when converted to estimated intervertebral angles (Fig. 4), are similar to those reported for lateral undulation and concertina in other species (Jayne, 1988a,b; Morinaga and Bergmann, 2019) and to our prior calculations based on lever arms (Tingle et al., 2023). By combining our results with previous electromyographic data on snake lateral undulation (Jayne, 1988b), we see that muscle activation and shortening occur synchronously, consistent with the idea that the muscles must produce net positive work to counteract losses via friction. The moving portions of the body during tunnel concertina also show this pattern (Jayne, 1988a). The slow relative shortening velocities observed in this study suggest that muscles operate over speeds which maximize force output rather than power during lateral undulation and tunnel concertina, though this inference will need to be confirmed with ongoing experiments measuring joint torques and muscle physiology.

A key limitation of this study is the lack of simultaneous electromyography, preventing definitive determination of whether observed shortening or lengthening is active or passive. However, previous work on the muscle activity patterns of lateral undulation in a congeneric species showed a highly stereotyped pattern of unilateral muscle activation that lets us infer muscle activation from body curvature: muscles on the right side of the body unilaterally activate when the body is maximally curved towards the left, and then remain active until the body is maximally curved to the right, at which point muscles on the left side of the body unilaterally activate (Jayne, 1988b). In combination with new data from our study, Jayne's (1988b) result suggests muscle function during lateral undulation consists almost entirely of concentric contractions. Muscle activity during concertina locomotion of a congeneric species is also associated with decreasing curvature of a given body section while it is moving, though this relationship does not hold for the portions of the body held static (Jayne, 1988a).

This study confirms the validity of our predictions for multiarticular muscle function based on mathematical modeling (Tingle et al., 2023), firmly establishing a baseline for a general understanding of the mechanics of multiarticular muscles. Because these equations use anatomical values that can be measured on animals beyond snakes (number of joints spanned, distance of the two attachment points from the axis of motion), they can serve as a starting point for a mechanistic understanding of function in numerous study systems. The most straightforward application of this mathematical framework will be to structures composed of serially repeated bones of similar shape and size, such as the tails or necks of many types of tetrapods. It might eventually be possible to adapt our mathematical model for more complex multiarticular systems, such as the regionalized vertebral columns of mammals.

## Supplementary Material

10.1242/jexbio.249259_sup1 Supplementary information

Table S1. This Excel file presents the results of statistical models. It has two sheets, one for lateral undulation and one for concertina.
