# Supplementary material for: Fluoromicrometry reveals minimal influence of tendon elasticity during snake locomotion
Source: J Exp Biol. 2025 Mar 3;228(5):JEB249259. doi: 10.1242/jeb.249259 (PMC11925394; doi:10.1242/jeb.249259)
Supplement: Supplementary information [file jexbio-228-249259-s1.pdf]

Table S1. This Excel file presents the results of statistical models. It has two sheets, one for lateral undulation and one for concertina.

Available for download at
https://journals.biologists.com/jeb/article-lookup/doi/10.1242/jeb.249259#supplementary-data

Supplementary Materials and Methods

Custom macro to smooth the backbone curve in ImageJ.

```
macro "Strikespline... [s]" {
     run("Fit Spline");
run("Measure");
length1 = getResult('Length', nResults-1);
length2=length1/128;
selectWindow("Results");
run("Close");
//print(length1);
//print(length2);
run("Interpolate", "interval=length2");
getSelectionCoordinates(x, y);
npts=x.length;
reflength=length1;
while (npts!=128) {
                run("Restore Selection");
                if (npts>128) {
                length1=length1-0.00001;
                }
                if (npts<128) {
                length1=length1+0.00001;
                }
                length2=length1/128;
                run("Interpolate", "interval=length2");
                getSelectionCoordinates(x, y);
                npts=x.length;
}
//print(npts);
run("Properties... ", "name=[] stroke=#4dffff00 width=20 list");
  }
```

Journal of Experimental Biology • Supplementary information
